# The paradox of autoimmunity and autoinflammation in inherited neutrophil disorders – in search of common patterns

**DOI:** 10.3389/fimmu.2023.1128581

**Published:** 2023-06-07

**Authors:** Damian Krzyzanowski, Aleksandra Oszer, Joanna Madzio, Maciej Zdunek, Julia Kolodrubiec, Bartosz Urbanski, Wojciech Mlynarski, Szymon Janczar

**Affiliations:** ^1^ Department of Pediatrics, Oncology and Hematology, Medical University of Lodz, Lodz, Poland; ^2^ Laboratory of Epigenetics, Institute of Medical Biology, Polish Academy of Sciences, Lodz, Poland

**Keywords:** neutropenia, chronic granulomatous disease, autoimmunity, inflammatory bowel disease, autoinflammation, severe congenital neutropenia

## Abstract

Congenital defects of neutrophil number or function are associated with a severe infectious phenotype that may require intensive medical attention and interventions to be controlled. While the infectious complications in inherited neutrophil disorders are easily understood much less clear and explained are autoimmune and autoinflammatory phenomena. We survey the clinical burden of autoimmunity/autoinflammation in this setting, search for common patterns, discuss potential mechanisms and emerging treatments.

## 1 Introduction

Autoimmunity has long been recognized as a sign of primary immunodeficiency disorders (PID) (1). Autoimmune disease might often be the initial or even the only clinical phenotype of inborn errors of immunity (1, 2). The paradigmatic PID with multisystem autoimmunity is *CTLA4* deficiency. The pathomechanism of autoimmunity in this monogenic disease is well-understood as CTLA4 is a major negative regulator of T cell activation in the immune synapse (3). There are further monogenic PIDs with pronounced autoimmunity such as for instance autoimmune polyglandular syndrome type 1, also called autoimmune polyendocrinopathy-candidiasis-ectodermal dystrophy (APS1/APECED) caused by pathogenic variants of *AIRE*, a transcription factor crucial in the process of the elimination of self-reactive T cells in thymus (4). An example of a more heterogenous clinically and genetically PID with frequent autoimmune phenomena is common variable immunodeficiency (CVID). In CVID and in monogenic syndromes reminiscent of CVID autoimmunity might be attributed to immune dysregulation in parallel and simultaneous to deficiency of the production of antibodies and effector cells, and appear to be mediated by compromised function or differentiation of B and especially T lymphocytes, including Tregs and other immunoregulatory lymphocyte subpopulations (5). While more universally, autoimmune comorbidities are not surprising and can be easily attributed to regulation failure in inborn defects of humoral or adaptive cellular immunity, the association of congenital defects of phagocyte number or function with autoimmunity is much more puzzling, likely multifactorial and more challenging to explain. These symptoms and comorbidities in phagocyte disorders are likely within a continuum of autoimmune and autoinflammatory processes as defined by dysfunction of the immune system resulting in the loss of immune tolerance against self-antigens, and driven by autoreactive T and B cells in ‘pure’ autoimmunity versus aberrant activation of innate immune system and inflammasomes in ‘pure’ autoinflammation (2, 3, 6–8).

The cellular and molecular events leading to autoimmunity in granulocyte disorders are much less well understood than in the defects of adaptive immunity. The existing and speculative explanations for increased autoimmunity and autoinflammation in inherited neutrophil disorders include in particular the consequences of excessive and prolonged infections, tissue injury, poor clearance of apoptotic cells (deficient efferocytosis), hyperactivation of inflammatory cytokines and eventually deficiency of regulatory cells similar to classical PIDs with autoimmunity with poor T cell homeostasis and loss of tolerance to self-antigens (4, 6). Frequent infections are leading to excessive antigen stimulation potentially with a degree of molecular mimicry between self and microbial antigens driving immune response against self-antigens (9). They may also trigger tissue and cellular injury leading to immune hyperactivation by pathogen- and damage-associated molecular patterns (PAMPs and DAMPs) (10). Another mechanism is a poor or non-physiological clearance of cellular debris that prolongs the exposure of immune cells to antigens and danger signals. In particular physiological efferocytosis that is an immune-privileged form of clearance of apoptotic cells is deficient in some neutrophil disorders (10). Efferocytosis normally not only compartmentalizes self-antigens but also provides local anti-inflammatory and immunosuppressive signals that prevent immune activation during apoptotic cell processing (11). Further, autoinflammatory cytokines might be excessively activated due to the processes mentioned above or as a direct effect of neutrophil defects or effects of monogenic defects in other cellular compartments apart from neutrophils similar to inflammasome errors in recurrent fever syndromes (2, 6). Finally, some reports point to qualitative or quantitative deficiency of regulatory cells such as Tregs in granulocyte disorders, the mechanisms of which are not clear and might be secondary to neutrophil dysfunction, metabolic defects or more direct consequences of gene mutations in T cell compartment rather than only myeloid cells (12–15).

We provide a structured overview of autoimmune and autoinflammatory phenomena in congenital defects of neutrophil number or function by presenting the specific and common clinical phenotypes, common and disease-unique mechanisms and emerging treatments, some of which are surprisingly specific considering the relatively less clear mechanisms as compared to ‘classic’ PID with autoimmunity.

## 2 Phenotypes

### 2.1 Chronic granulomatous disease

Chronic granulomatous disease (CGD) is caused by autosomal and X-linked mutations in nicotinamide adenine dinucleotide phosphate (NADPH) oxidase complex genes (16). While the most important features of CGD are susceptibility to fungal and bacterial infections as well as less well explained formation of granulomas, it is also associated with a high prevalence of inflammatory and autoimmune phenomena that are estimated to be seen in up to 70% of CGD patients. Among these the most frequently reported with a rate of around 50% are gastrointestinal symptoms. They range from chronic abdominal symptoms such as pain, nausea, and diarrhea, to more severe conditions like fistulae, strictures, and inflammatory bowel disease that meet the criteria for Crohn’s disease, supported by consistent biopsy histopathological findings. According to some authors severe inflammatory bowel disease (IBD) is more frequent in X-linked CGD than in autosomal forms, but other reports did not confirm that. Testing for CGD is warranted in all patients with very early onset IBD (6, 17–21). Further, a significant proportion of patients presents various non-infectious pulmonary morbidity, including at its severe end of spectrum interstitial lung disease. Some infectious pulmonary complications are characterized by ‘exaggerated’ inflammatory phenotype, including notorious mulch pneumonitis. Further, a variable between the studies but significant proportion of patients present systemic autoimmune or inflammatory disease complicating CGD. These rheumatologic/autoimmune phenomena include: systemic lupus erythematosus (SLE), discoid cutaneous lupus, vasculitides, dermatomyositis, juvenile idiopathic arthritis and antiphospholipid syndrome and overlapping clinical forms. Organ specific autoimmunity include immune thrombocytopenia and IgA nephropathy (6, 17–20, 22, 23).

There is a surprising but growing and well established link between CGD and hemophagocytic lymphohistiocytosis (HLH). A CGD-related gene variant is the most frequent genetic finding in children meeting clinical HLH criteria negative for classical HLH-gene defects. A CGD-associated HLH episode is not always overtly infection-triggered and might by the first clinical situation bringing patients with CGD to medical attention (6, 24–31). We suggest that CGD genes should be included in extended HLH gene panels if pathogenic variants in ‘classical’ familial hemophagocytic lymphohistiocytosis (FHL) genes are not found.

Interestingly, hypomorphic variants in CGD-associated genes, most of these with minimal impact on NADPH activity, were identified as genetic risk factors for a number of autoimmune and autoinflammatory diseases (6).

### 2.2 CGD female carriers

Female carriers of X-linked CGD have a dual phagocyte population, as a consequence of lyonization, with varied proportion of functioning granulocytes. While they are usually free of infectious complications, there is a surprisingly high prevalence of autoimmune and inflammatory manifestations. This suggests that a proportion of dysfunctional granulocytes is enough to provoke these phenomena. Reported morbidity includes in particular gastrointestinal symptoms sometimes fulfilling the criteria of inflammatory bowel disease, but more frequently described as unspecific colitis or persistent diarrhea. Other described clinical association of CGD carrier status is discoid lupus erythematosus and related symptoms such as photosensitivity, oral ulcers, arthritis, Raynaud phenomenon and alopecia (32–34).

### 2.3 Leukocyte adhesion deficiency type-1

Leukocyte adhesion deficiency type-1 (LAD-I), caused by mutations in ITGB2 (also known as LFA-I) integrin leading to poor neutrophile chemotaxis, is apart from other symptoms and complications, frequently characterized by severe periodontitis and this is believed to be mediated by excessive activation of an inflammatory axis rather than a simple microbial complication (35). The mechanism behind this phenomenon is discussed below. Inflammatory bowel disease is also reported in LAD-I (36, 37).

No associations for other leukocyte adhesion syndromes and autoimmunity/autoinflammation was described.

### 2.4 Severe congenital neutropenia

Severe congenital neutropenia (SCN) is a group of rare genetic disorders characterized by a significant decrease in the number of neutrophils in peripheral blood. The condition is caused by mutations in several genes, including *ELANE*, *HAX1*, *SLC37A4*, *G6PC3*, and many others (38, 39). *ELANE* gene mutations are the most common cause of SCN and account for around 60% of cases (40).

#### 2.4.1 SCN-4 (Glucose-6-Phosphatase Catalytic Subunit 3 deficiency)

Glucose-6-Phosphatase Catalytic Subunit 3 (G6PC3) deficiency is an ultrarare disease so large series of patients are lacking. The data from the limited number of patients with this disease point to a relatively high frequency of immune thrombocytopenia (ITP) and, frequently of severe clinical course, inflammatory bowel disease. IBD in SCN-4 might be non-responsive to G-CSF but improve after TNFα blockage (41–45). There are also reports of an autoinflammatory phenotype in G6PC3-deficiency with systemic amyloidosis (46) or arthritis and mucosal/gingival lesions (47).

#### 2.4.2 Glycogen storage disease type 1b

Glycogen storage disease type 1b (GSD type 1b) is a autosomal recessive disease caused by a mutation in the glucose-6-phosphate transporter gene (*G6PT1*, also known as *SLC37A4*). The clinical hallmarks of the disease are hypoglycemia and other metabolic disturbances, dysfunction of the liver and kidneys, seizures, various organ complications and chronic neutropenia. This catalogue of morbidities was recently extended by the realization of the high frequency of autoimmune manifestations. These include IBD, and less frequently thyroid autoimmunity and myasthenia (12, 14, 15, 48, 49). This organ-specific autoimmunity in GSD type 1b is in a way standing out from the other described phenotypes in neutrophil disorders that usually have a significant autoinflammatory component. Nevertheless, there are also reports of an autoinflammatory phenotype in GSD type 1b with renal or systemic amyloidosis (50, 51).

#### 2.4.3 ELANE-SCN and cyclic neutropenia

The patients with severe congenital neutropenia caused by mutations in the gene coding neutrophil elastase (*ELANE*) and especially cyclic neutropenia (CyN) present recurrent periodontitis and fever episodes, whose severity cannot be fully explained as an infectious complication. They bear clinical similarity to autoinflammatory disorders (periodic fever symptoms), especially in the case of CyN. There is a report of elevated level of the major autoinflammatory cytokine IL-1β in gingival crevicular fluid of patients with SCN-ELANE in a study of the correlations of periodontitis in SCN (52). To our knowledge, parallel data on IL-1β has so far never been obtained in serum of patients with CyN or SCN-ELANE. Also, linking SCN and especially CyN with recurrent fever syndromes are the descriptions of amyloidosis, typical for uncontrolled autoinflammatory disorders, in ELANE-mutant SCN. These include especially reports of both renal and systemic amyloidosis (53–56). Recently, our group reported that ELANE-SCN patients have a high risk of developing antibodies against neutrophils. While the mechanism and significance is not clear it is the first evidence of autoantibodies in ELANE-SCN/CyN (57). **Table 1** summarizes phenotypes across major entities/syndromes discussed above (CGD, CGD carriers, LAD and various forms of SCN).

**Table 1:** The summary of associations of autoimmune and autoinflammatory phenotypes/complications in inherited neutrophil disorders.

Phenotype /Disease	CGD	XL-CGD	LAD-I	G6PC3-deficiency	GSD1b	ELANE-SCN	CyN
**SLE**							
**JIA**							
**Arthritis**							
**Vasculitides**							
**APS**							
**IBD**							
**DM**							
**ITP**							
**Thyroid**							
**Myasthenia**							
**ILD**							
**Hepatitis**							
**Gingivitis**							
**HLH**							
**Amyloidosis**							

CGD, Chronic granulomatous disease; XL-CGD, X-linked CGD carrier; LAD-I, Leucocyte adhesion deficiency type-1; GSD1b, Glycogen storage disease type 1b; CyN, Cyclic neutropenia.

SLE, systemic lupus erythematosus; JIA, juvenile idiopathic arthritis; APS, antiphospholipid syndrome; IBD, inflammatory bowel disease; DM, diabetes mellitus; ITP, immune thrombocytopenia; Thyroid, thyroid autoimmunity; ILD, inflammatory/interstitial lung disease; HLH, hemophagocytic lymphohistiocytosis. List of references: CGD and X-linked CGD (6, 17, 20, 21, 32); LAD-I (35, 37); G6PC3-deficiency (44, 46); GSD1b (12, 14, 48); ELANE-SCN and CyN (52, 54, 55).

## 3 Mechanism

### 3.1 Common mechanism

While there is a surprising convergence of the observed autoimmune/autoinflammatory symptoms, common mechanisms for increased autoimmunity and autoinflammation in inherited neutrophil disorders remain speculative, i.e. the existent data are usually on disease-specific processes. Common mechanism can be proposed to include prolonged and poorly controlled infections leading to excessive antigen stimulation potentially with a degree of molecular mimicry between self and microbial antigens driving immune response against self-antigens. This is likely associated with tissue injury, poor clearance of cellular debris resulting in signals from pathogen- and damage-associated molecular patterns and finally hyperactivation of inflammatory cytokines (6, 7, 9). In fact, while likely, the role and impact of infections on autoimmunity is not well formally studied in the context of neutrophil disorders. There are however data suggesting that better infection control impacts positively on non-infectious complications (6, 58).

### 3.2 Specific mechanisms

#### 3.2.1 CGD

**Figure 1:**
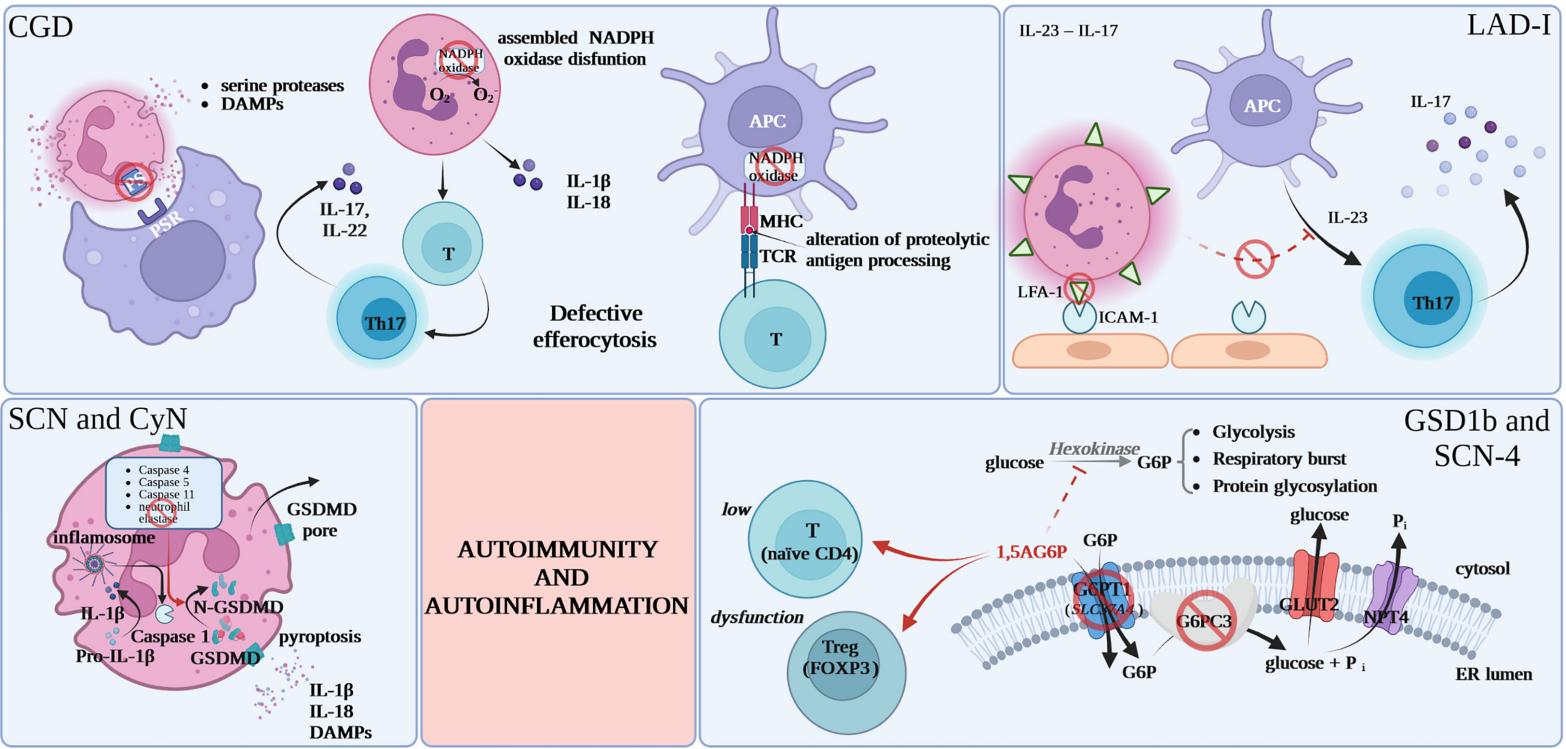
Proposed common mechanisms for increased autoimmunity and autoinflammation in inherited neutrophil disorders. CGD, chronic granulomatous disease; DAMPs, damage-associated molecular patterns; PS, phosphatidylserine; PSR, specific phosphatidylserine receptor; APC, antigen presenting cell; MHC, major histocompatibility complex; TCR, T-cell receptor; NADPH, nicotinamide adenine dinucleotide phosphate; GSDMD, gasdermin D; FOXP3, forkhead box P3; 1,5AG6P, 1,5-anhydroglucitol-6-phosphate; G6P, glucose 6-phosphate; G6PC3, glucose-6-phosphatase, catalytic 3; Pi, inorganic phosphate; GLUT2, glucose transporter 2; NPT4, solute carrier family 17 (organic anion transporter), member 3; ER, endoplasmic reticulum.

While the major mechanism behind autoimmune and autoinflammatory phenomena in CGD appears to be deficient efferocytosis of apoptotic cells by CGD-phagocytes the contributing mechanisms remain elusive as CGD is genetically heterogeneous and cells of different lineages are dysfunctional as discussed below (as summarized in **Table 2**). The processing of self-antigens after “physiological” apoptosis is followed by the exteriorization of phosphatidylserine (PS) by apoptotic cells, which are recognized through specific receptors on macrophages. This is an immunoprivileged cell death pathway primarily through protection against excessive premature membrane disintegration and exposure to proteases and oxidizing enzymes. Deficient efferocytosis leads to prolonged and increased DAMP stimulation and loss of local anti-inflammatory signal. Presentation of self-antigens associated with ‘danger’ signals from necrotic cells or apoptotic cells without efficient efferocytosis leads to potential breakage of immune tolerance (10). While deficient efferocytosis (or other danger signal-related mechanisms) is a very plausible common phenomenon in genetically diverse CGD subtypes and is likely the major mechanism contributing to autoimmunity/autoinflammation in CGD this explanation for autoimmune, and especially autoinflammatory, phenomena in CGD remains speculative and that would be very difficult to provide direct data (**Figure 1**).

**Table 2:** The overview of the congenital neutrophil disorders presented in this review (modes of inheritance, affected genes, prevalence, affected cell populations and overview of the potential mechanisms of autoimmunity/autoinflammation).

Disease	Mode of inheritance	Affected genes	Prevalence	Affected cellular populations	Potential mechanisms
**CGD**	X-linked, AR	*CYBA, CYBB, G6PD, NCF1, NCF2, NCF4*	1:200 000	neutrophils, eosinophils, monocytes, macrophages, T lymphocytes, B lymphocytes	deficient efferocytosis, increased danger signaling, intrinsic T defects, intrinsic B defects, antigen presentation dysregulation
**LAD-I**	AR	*ITGB2*	1:10 0000 000	neutrophils, lymphocytes	IL-23 – IL-17 cytokine axis dysregulation
**G6PC3-deficiency**	AR	*G6PC3*	1:2 500 000 to 1:5 000 000	neutrophils, T lymphocytes	deficient efferocytosis, increased danger signaling, intrinsic T lymphocyte defects
**GSD1b**	AR	*G6PT1/SLC37A4*	1:500 000 to 1:2 000 000	neutrophils, T lymphocytes	deficient efferocytosis, increased danger signaling, intrinsic T defect
**ELANE-SCN**	AD	*ELANE*	1:500 000	neutrophils	increased danger signaling, aberrant cytokine signaling – speculative
**CyN**	AD	*ELANE*	1:1 000 000	neutrophils	increased danger signaling, aberrant cytokine signaling – speculative

CGD, Chronic granulomatous disease; CYBA, cytochrome B-245 alpha chain; CYBB, cytochrome B-245 beta chain; G6PD, glucose-6-phosphate dehydrogenase; NFC1, neutrophil cytosolic factor 1; NFC2, neutrophil cytosolic factor 1 (59–62); LAD-I: ITGB2, integrin subunit beta 2 (63, 64); G6PC3-deficiency: G6PC3, glucose-6-phosphatase catalytic subunit 3 (65); GSD1b: SLC37A4, solute carrier family 37 member 4 (62, 66); ELANE-SCN and CyN: ELANE, neutrophil elastase (62).

NADPH deficiency was also postulated to lead to intrinsic T lymphocyte defects, which may result, at least speculatively, in the initiation of more classical T-cell dependent autoimmunity. Another postulated mechanism is the impact of the loss of ROS products of NADPH on intracellular signaling pathways including in particular NF-κB, MAPKs, hypoxia-regulated pathways. Also, the defects of NADPH oxidase are potentially altering antigen presentation by MHC molecules *via* alteration of proteolytic antigen processing. Other authors believe that some of CGD comorbidities of both infectious and non-infectious character are caused by the dysregulation of indoleamine-2,3-doxygenase, which is an immunoregulatory enzyme in the tryptophan catabolic pathway, or improper Th17 signaling. There are also reports of increased release of autoinflammatory cytokines i.e. IL-1β and IL-18 by CGD-neutrophils (7, 8, 13, 21), increased interferon signatures in patients peripheral blood leukocytes (67) as well as increased TNFα and IL1β signaling in CGD-monocytes (68–71). Further, building up the complexity, there are also data on B cell maturation dysregulation in CGD potentially contributing to autoimmunity (72, 73). It is hard to conclude whether these alterations are causative or co-existent as a consequence of CGD-associated inflammation.

To make the situation even more complex, there is a growing number of reports on the contribution of hypomorphic variants in CGD-associated genes, usually with minimal impact on NADPH oxidase activity, to a number of autoimmune and autoinflammatory diseases. This drives the conclusion that the mechanisms of autoimmunity/autoinflammation in CGD might involve various pathways both directly related and potentially unrelated to NADPH oxidase activity (6, 74).

#### 3.2.2 XL-CGD female carriers

The mechanisms are extremely likely the same as in CGD, however the presence of symptoms in many carriers point to the fact that a proportion of deficient phagocytes might be enough to cause syndromes (32, 33, 75, 76). This may suggest that the symptoms are due to proinflammatory signals or cytokines produced by some oxidase-deficient myeloid cells rather than complete lack of efficient cells that might fight infections or clear tissue debris.

#### 3.2.3 Leukocyte adhesion deficiency type-1

New reports from preclinical models point to IL-23 – IL-17 cytokine axis imbalance as a mechanism behind severe periodontal disease in LAD-I. Lack of tissue neutrophils leads to dysregulation of the IL-23 pathway, causing excessive IL-23 and IL-17 responses at barrier sites like the oral mucosa, skin, and gastrointestinal tract. In normal conditions there is a subsequent down-regulation of IL-23 as well as downstream cytokines: IL-17 and granulocyte colony-stimulating factor (G-CSF) production (77, 78). Due to the low amount of tissue neutrophils in LAD-I, the normal circuit is disrupted, leading to an overreaction of interleukin-23 and interleukin-17, which may be responsible for the immunopathological processes specific to LAD-I in the affected area (79, 80). There are reports of periodontal inflammation resolution in LAD-I patient treated with a dual antibody against IL-23/IL-17 (6, 35–37, 80, 81).

#### 3.2.4 SCN

##### 3.2.4.1 Glycogen storage disease type 1b and SCN-4 (G6PC3-deficiency)

Neutropenia and neutrophil dysfunction in GSD type 1b and SCN-4 were recently shown to be the result of 1,5-anhydroglucitol-6-phosphate (1,5AG6P) accumulation in granulocytes caused by the loss of G6PT1 or G6PC3 that normally collaborate to destroy this metabolite. 1,5AG6P reaches concentrations that strongly inhibit hexokinase activity and lead to poor glucose utilization and cell death (82). Consistent with that neutrophil loss in GSD type 1b is believed to be caused by excessive apoptosis rather than deficient maturation (83). Apart from increased rates of apoptosis, GSD type 1b-neutrophils display compromised respiratory burst, chemotaxis, and calcium mobilization (84). While this is less well documented in G6PC3-deficiency common mechanism and patterns are very likely for the two diseases and this view is supported by the utility of similar new therapeutic approaches as discussed below. Consistently, recent literature points out to functional defects of respiratory burst and chemotaxis in granulocytes caused by the metabolic disturbance in G6PC3-deficiency (85).

The poor cellular glucose utilization that results in neutrophil dysfunction and loss also seems to be relevant in the lymphoid compartment and causative for T cell defects leading to autoimmunity in GSD type 1b and G6PC3-deficiency. In GSD type 1b this is reflected by lymphopenia and the reduction in FOXP3+ regulatory T cell (Tregs) function and in G6PC3-deficency by CD4-lymphopenia and loss of thymic naïve CD4 cells. These T cell abnormalities are likely causative for organ-specific autoimmunity (12, 42).

Interestingly another abnormality common in GSD type 1b and G6PC3-deficiency is reported to be hypo-glycosylation of gp91 (phox), the electron-transporting component of the NADPH oxidase (86). This provides and an interesting similarity to CGD and may partly explain the strong association of this disease with IBD and more broadly with autoinflammation due to mechanisms similar to those acting in CGD and discussed above. This autoinflammatory phenotype leading to IBD in G6PC3-deficiency was highlighted in a recent publication (41).

##### 3.2.4.2 ELANE-SCN and CyN

Similarly to LAD-I, severe periodontitis seen in CyN or SCN-ELANE might be mediated by excessive activation of an inflammatory axis rather than only microbial complication. As mentioned above ELANE-SCN/CyN might be associated with amyloidosis, pointing to an autoinflammatory component. There are practically no studies of IL-1β, SAA or other autoinflammatory markers in SCN. Interestingly, ELANE is reported to be one of the few proteins processing gasdermin D, which is among significant major players in pyroptosis and in IL-1β production regulation (87, 88). ELANE mutations might be expected to impact GSDMD cleavage or intracellular trafficking. Gasdermin D activity or processing was not studied so far in the context of mutated ELANE or SCN. Interestingly, gasdermin D is differentially regulated in SCN (89).

## 4 Potential and emerging interventions

### 4.1 General approaches

#### 4.1.1 Infection control

Appropriate infection control specific for the disease such as antibacterial and antifungal prophylaxis in CGD or G-CSF in congenital neutropenias limits also the extent of inflammatory complications and their sequelae (2, 58, 90).

#### 4.1.2 Allogeneic hematopoietic stem cell transplantation (alloHSCT)

AlloHSCT is increasingly becoming a standard treatment in CGD, apart from antimicrobial prophylaxis. This is usually performed unless the phenotype is very mild, there are specific contraindications or the patient or the guardian give no consent for transplantation. While in general HSCT is performed because of the life-threatening and associated with severe complications and sequelae infections, there are numerous reports showing that HSCT also rescues the autoimmune/autoinflammatory phenotype (6, 21, 91).

aHSCT is also the only curative therapeutic approach in LAD-I. While this is done to avoid morbidity and mortality associated with severe infections, it also seems to reduce non-infectious complications (35, 92).

In severe congenital neutropenias (ELANE-SCN, CyN, G6PC3-deficiency, GSD type 1b) HSCT is performed in case of G-CSF refractoriness or Myelodysplastic syndromes (MDS)/acute myeloid leukemia (AML) transformation. The latter is very rare in the entities discussed here apart from ELANE-SCN (90). Similarly for CGD, however with low patient numbers reported, there is evidence for amelioration or complete correction of the autoimmune/autoinflammatory phenotype, mostly described for IBD in G6PC3-deficiency and GSD type 1b (43).

#### 4.1.3 Gene therapy

Gene therapy is plausible and/or actively investigated in preclinical models and in clinical studies in CGD, LAD type 1 and GSD type 1b (21, 58). The positive or curative impact on non-infectious complication is very likely, but so far minimal data is available.

#### 4.1.4 Immunosuppression and anticytokine drugs

Immunosuppression or ‘biologic’ (anticytokine) drugs are administered as indicated by standard treatment in a recognized particular autoimmune/autoinflammatory disease. Still, it must be considered that both immunosuppressant and anticytokine drugs may exacerbate the infectious phenotype in neutrophil disorders. In particular, there is a concern that anti-TNF drugs may increase the risk of invasive aspergillosis in CGD, and they are generally contraindicated (6, 17, 21, 93).

There are varied reports on the clinical utility of immunosuppressive and anticytokine drugs in autoimmunity associated with congenital neutrophil disorders. In general, the cohorts of patients are of very low numbers and not subjected to any form of controlled trails. Some reports claim their clinical effectiveness might be lower than in other IBD patients, others do not confirm this (6, 93).

We are not aware of any attempts of anticytokine drug use in periodontitis or recurrent fevers associated with ELANE-SCN or CyN.

Hemophagocytic lymphohistiocytosis (HLH)/macrophage activation syndrome (MAS) appearing in the setting of CGD does not generally have a fulminant course typical for classical familial HLH and can usually be treated with steroids, intravenous immune globulin (IVIG) and occasionally cyclosporine A without full HLH protocol with etoposide (6).

### 4.2 Specific approaches

#### 4.2.1 Pioglitazone in CGD

Pioglitazone, an antidiabetic used in type II diabetes, which is a peroxisome proliferator-activated receptor γ (PPARG) agonist, increases mitochondrial oxygen species production and killing activity of CGD-granulocytes and was shown to improve the efficiency of efferocytosis in preclinical models. Recently, there are first clinical data showing both functional effects and ameliorated symptoms/disease course in CGD patients treated with pioglitazone. This drug might be used for example to ameliorate the symptoms before HSCT. Some reports question its clinical utility in CGD (21, 94).

#### 4.2.2 SGLT2 inhibitors in GSD type 1b and G6PC3-deficiency

The appreciation of the common neutrophil failure mechanism in GSD type 1b and G6PC3-deficiency, lead to the preclinical attempts at 1,5AG6P reduction. This was achieved by treatment with an inhibitor of renal glucose transporter Sodium-glucose co-transporter-2 (SGLT2) in murine models with spectacular effect on neutrophil counts. Shortly afterwards, this approach was successfully attempted in patients with GSD type 1b disease using off-label empagliflozin (15, 95). Re-purposing of this antidiabetic drug is an impressive example of mechanism-driven medical intervention and seems to improve neutropenia and reduce G-CSF consumption as well as to improve other phenotypes in GSD type 1b. While SGLT2-inhibition seem to ameliorate IBD the impact on other and especially organ-specific autoimmune complications remains to be determined (95–99). Recently, empagliflozin also showed promising results in 2 patients with G6PC3-deficiency (100).

Prospective studies of SGLT2 inhibitors in larger groups of children with GSD1b and/or G6PC3 are currently in progress, but the results are not yet available (ClinicalTrials.gov Identifiers: NCT04986735, NCT04138251, NCT05078879).

## 5 Conclusions

Autoimmune/autoinflammatory diseases must be considered as frequent and important comorbidities in congenital defects of neutrophil number and/or function. This not always satisfactorily appreciated by the medical community and we believe the awareness of such symptoms, syndromes and their causes should be improved. The mechanisms behind this complications are still not satisfactorily explained, and include both common as well as disease-specific patterns. Given the widely varied molecular and cellular mechanisms behind autoimmune/autoinflammatory phenomena there is a surprising clinical convergence with especially frequent presence of inflammatory bowel disease in many of the presented diseases (6, 44). This may support the view that the leading mechanisms are poorly controlled infections and poor clearance of cellular debris by phagocytes resulting in strong and persistent immune activation and cytokine production. It must be acknowledged, however, that the available literature enumerates and investigates the potential mechanisms, but there are no conclusions regarding their relative impact on the observed phenotypes and it is frequently not clear if studied immune abnormalities are causative or merely associated with the disease. While some new and mechanism-driven treatments are emerging (14, 100, 101), there is a lack of consensus or standard diagnostic and therapeutic approaches in autoimmunity associated with neutrophil disorders. There are doubts whether the comorbidities such as for example Crohn’s disease should be regarded as bona fide IBD or rather intestinal manifestation of CGD or a similar immune defect (6, 102). Further, there are controversies about the potential effect of immunosuppression or anticytokine drugs on infection risk and on how to balance their use with infection risk (93). On the other hand uncontrolled autoimmunity elevates the risk for any morbidities including infections. These difficulties and doubts should stimulate further research into mechanistically driven treatments or gene therapy to limit the use of non-specific interventions on the immune system. Apart from mechanistic studies, larger series of patients with monitoring of autoinflammatory markers such as IL-1β, IL-18 or SAA, would be helpful in understanding the burden of autoinflammation in congenital neutrophil disorders and perhaps for establishing therapeutic windows for broader anticytokine intervention. Speculatively, anti-IL-1 treatments could be used for the control of periodontitis in for example CyN and such interventions could also interrupt the periodic granulopoiesis dysfunction in CyN if that is at least partly driven by cytokine effects on hematopoiesis in bone marrow. Finally, HSCT, performed because of uncontrolled infections or MDS/AML transformation, might be curative for non-infectious morbidity including autoimmune disease.

## Author contributions

DK reviewed and evaluated the literature, drew the figures, designed and edited drafts of the manuscript and finalized the manuscript for submission. AO reviewed the literature and prepared drafts of the manuscript. JM reviewed the literature, prepared the table and prepared drafts of the manuscript. MZ, JK, BU reviewed the literature and prepared drafts of the manuscript. WM and SJ contributed to the conception and provided input throughout, designed and edited drafts of the manuscript. All authors contributed to the article and approved the submitted version.
